# Editorial: Immune Responses in Sexually Transmitted Infections Caused by Parasites and DNA Viruses: New Insights

**DOI:** 10.3389/fcimb.2022.838799

**Published:** 2022-01-27

**Authors:** Manisha Yadav, Nancy Malla

**Affiliations:** ^1^Dr. B.R. Ambedkar Center for Biomedical Research, University of Delhi, Delhi, India; ^2^Department of Medical Parasitology, Post Graduate Institute of Medical Education and Research (PGIMER), Chandigarh, India

**Keywords:** *Trichomonas vaginalis*, sexually transmitted diseases, DNA viruses, immune responses, HPV

## Introduction

Sexually transmitted infections (STIs) caused by sexually transmissible pathogens affect more than 300 million people annually. More than 30 sexually transmitted pathogens which include bacteria, parasites, and viruses are known. Human trichomoniasis is the most common non-viral STI caused by *Trichomonas vaginalis* (TV), a protozoan parasite. STIs caused by viruses include herpes simplex virus (HSV), hepatitis A & B virus, human papillomavirus (HPV), and cytomegalovirus. If these infections remain untreated, it may lead to severe complications, significant morbidity, and mortality.

Although much literature is available on STIs, different aspects need further exploration especially in terms of immune factors for early diagnosis and effective treatment. Innate and adaptive immune responses affect the establishment of and contribute to pathology. The role of innate and adaptive immunity in vaccine development and the role of cell-mediated responses in the genital tract are under investigation. Nod-like receptors (NLRs), Toll-like receptors (TLRs), and T-regulatory (Tregs) cells have been investigated. The role of TLRs in immune responses to Trichomonas infections is well documented (Yadav et al., 2021). In this Research Topic, we discuss the role of different TLRs in various STIs caused by viral, bacterial, and protozoan pathogens, focusing on specific ligands and signaling by TLRs. We also discuss the role of single nucleotide polymorphisms (SNPs) of TLRs in STIs.

The aim of the Research Topic entitled “*Immune Responses in Sexually Transmitted Infections Caused by Parasites and DNA Viruses: New Insights*” is to provide details of the different aspects of STIs caused by parasites and DNA viruses with special focus on immune responses for healthcare professionals interested in these infections.

Under this Research Topic, five publications contribute updated knowledge on this subject. The research article entitled ‘*Protozoan-Viral-Bacterial Co-Infections Alter Galectin Levels and Associated Immunity Mediators in the Female Genital Tract*’ (Fichorova et al.) details a new role of galectins, a glycan-sensing family of proteins, in mixed infections. The authors assessed participants in the HIV Epidemiology Research Study at each of their incident visits matched to controls who remained *Trichomonas vaginalis* (TV) negative throughout the study. Incident TV was associated with higher levels of galectin-1, galectin-9, IL-1β, and chemokines. Galectin-9, IL-1β, and chemokines were up and galectin-3 down in TV cases with bacterial vaginosis (BV) or intermediate Nugent *versus* normal Nugent scores (p <0.001). Galectin-9, IL-1β, and chemokines were increased in TV-HIV and reduced in TV-HPV co-infections. *In-vitro*, TV synergized with its endosymbiont *Trichomonas virus* (TVV) and *Prevotella bivia* (BV) bacteria to upregulate galectin-1, galectin-9, and inflammatory cytokines. The BV-bacterium alone, and together with TV, downregulated galectin-3 and synergistically upregulated galectin-1, galectin-9, and IL-1β, suggesting galectin-mediated immunity may be dysregulated and exploited by viral-protozoan-bacterial synergisms exacerbating inflammatory complications from dysbiosis and STIs. Further studies are required in diverse populations to address the role of socio-demographic factors and health disparities in galectin-mediated immunity underlying susceptibility to mixed protozoan-viral-bacterial infections.

The review article titled ‘*The Immune Microenvironment in Human Papilloma Virus-Induced Cervical Lesions - Evidence for Estrogen as an Immunomodulator*’ (Jayshree R. S.) describes the local immune responses in human papillomavirus (HPV) infection of the uterine cervix. Persistent infection by high risk HPV genotypes is assisted by other risk factors in the progression through precancer (various grades of cervical intraepithelial neoplasia-CIN) to cervical cancer (CxCa). During the gradual evolution of CIN 1 to CxCa, the microenvironment of the lesions undergo the three ‘Es’ of cancer immunoediting, viz., elimination, equilibrium, and escape. Both cell-intrinsic and extrinsic mechanisms operate in eliminating virally infected/precancerous cells. Amongst the cell-extrinsic players, which converge into the lesions, are varied types of anti-tumor/antiviral effectors like the Th1 subset of CD4+ T cells, CD8+ cytotoxic T cells, natural killer cells, etc., and pro-tumorigenic/immunosuppressive cells like regulatory T cells (Tregs), myeloid-derived suppressor cells (MDSCs), type 2 macrophages, etc. The final outcome of the infection/precancer nevertheless is dependent on which of these cell populations gain an upper hand in the lesion. Estradiol has been well recognized to be a co-factor in cervical carcinogenesis. The review highlights the role played by elevated tissue levels of the hormone as a growth factor for cervical epithelial cells and more importantly also as a potentiator of the function of various stromal and infiltrating immunosuppressive cells, viz., Tregs, MDSCs, and carcinoma-associated fibroblasts operating *via* estrogen receptor-α. The immunomodulatory role of estradiol in HPV-mediated cervical lesions is reviewed.

Another compelling study entitled ‘*Genotype Distribution Change After Human Papillomavirus Vaccination in Two Autonomous Communities in Spain*’ (Freire-Salinas et al.) has addressed the need to change the screening strategy of uterine cervical cancer. Screening based on molecular tests is desired, and distribution of the HPV genotypes after the introduction of the vaccination program with Cervarix^®^ and Gardasil 4^®^ in two autonomous communities in Spain, looking for possible changes in distribution and the occurrence of a herd effect, is described. In this study, populations before and after implementation of the HPV vaccine were recruited from the same geographic locations and category of service provider to minimize changes in the HPV risk-related characteristics between the two periods. Three HPV types that are genetically related to the vaccine-targeted HPV types, i.e., HPV45 (related to HPV18) and HPV31 and HPV33 (related to HPV16), against which cross-protective antibodies have been detected in response to HPV vaccination. Genotypes HPV6 and HPV11 have decreased significantly after the introduction of the vaccine. HPV16 has also decreased, but not significantly. However, the percentage of infection by HPV31, HPV52, and HPV45 has increased. In conclusion, continued surveillance is needed to provide further indication of any changes over time in the genotypes in circulation. This will be facilitated by the monitoring of the genotyping results from the new model of cervical screening using primary HPV DNA testing.

The research article entitled ‘*Role of Epstein-Barr Virus and Human Papillomavirus Coinfection in Cervical Intraepithelial Neoplasia in Chinese Women Living With HIV*’ (Feng et al.) highlights the prevalence of EBV and HPV coinfection in HIV-positive women and explored modulation of epithelial gene expression due to abnormal host immune status induced by viral coinfections. The authors found a significant correlation between EBV-HPV coinfection and the incidence of high-grade cervical intraepithelial neoplasia (CIN2+). The authors reported transcriptional changes in pathological tissues from HIV-positive women with EBV and HPV coinfection. These findings provide some evidence that EBV can act as a cofactor or mediator in HPV-related cervical cancer. Specific genes or proteins, such as CACNG4, may serve as biomarkers that can risk-stratify patients based on pathological changes in the cervix. The relatively small sample size is the limitation of the study. Further large-scale studies of the general population are required to validate and select specific biomarkers for early intervention in cervical cancer.

Finally, the review article entitled ‘*Localized and Systemic Immune Response in Human Reproductive Tract*’ (Gudisa et al.) details STIs and host immune response. STIs remain under-reported, and many infections have taken an epidemic turn. The biggest roadblock in this is the unknown basis of immunoprotective targets for these infections, hindering the discovery of potential targets for immunization. Authors discuss the complex interplay between innate immune defenses, with resident microbiota and mucosal immune responses serving as the basis for therapeutic approaches, by targeting the vital steps of this dynamic interaction. The characterization of pathogen-specific antibodies to significant immunogenic molecules may reveal the potential protective targets.

In view of the facts mentioned above, this Research Topic was launched to discuss the latest trends in the field and to discuss the possibilities of tweaking an effector arm of immunity for controlling the pathogen burden in host cells. Several leading groups across the globe have contributed to this Research Topic and covered various intriguing aspects of signaling including host pathogen interaction during an acute infection in macrophages.

## Conclusion and Perspective

The focused research and review articles under this proposal renders this Research Topic a successful collection of key findings that may shed new light on various intriguing aspects of STIs caused by parasites and DNA viruses. The publications provide valuable information and updated knowledge to the healthcare professionals to understand in detail the different aspects of STIs caused by parasites and DNA viruses with special focus on immune responses.

## Author Contributions

All authors contributed to the article and approved the submitted version.

## Funding

MY is a recipient of the ICMR-DHR international fellowship.

## Conflict of Interest

The authors declare that the research was conducted in the absence of any commercial or financial relationships that could be construed as a potential conflict of interest.

## Publisher’s Note

All claims expressed in this article are solely those of the authors and do not necessarily represent those of their affiliated organizations, or those of the publisher, the editors and the reviewers. Any product that may be evaluated in this article, or claim that may be made by its manufacturer, is not guaranteed or endorsed by the publisher.
